# Cadaverine Is a Switch in the Lysine Degradation Pathway in *Pseudomonas aeruginosa* Biofilm Identified by Untargeted Metabolomics

**DOI:** 10.3389/fcimb.2022.833269

**Published:** 2022-02-14

**Authors:** Abigail Leggett, Da-Wei Li, Devin Sindeldecker, Amelia Staats, Nicholas Rigel, Lei Bruschweiler-Li, Rafael Brüschweiler, Paul Stoodley

**Affiliations:** ^1^ Ohio State Biochemistry Program, The Ohio State University, Columbus, OH, United States; ^2^ Department of Chemistry and Biochemistry, The Ohio State University, Columbus, OH, United States; ^3^ Department of Microbial Infection and Immunity, The Ohio State University, Columbus, OH, United States; ^4^ Campus Chemical Instrument Center, The Ohio State University, Columbus, OH, United States; ^5^ Biomedical Sciences Graduate Program, The Ohio State University, Columbus, OH, United States; ^6^ Department of Microbiology, The Ohio State University, Columbus, OH, United States; ^7^ Department of Biological Chemistry and Pharmacology, The Ohio State University, Columbus, OH, United States; ^8^ Department of Orthopaedics, The Ohio State University, Columbus, OH, United States; ^9^ National Biofilm Innovation Centre (NBIC) and National Centre for Advanced Tribology at Southampton (nCATS), Mechanical Engineering, University of Southampton, Southampton, United Kingdom

**Keywords:** biofilm, *Pseudomonas aeruginosa*, NMR-based metabolomics, cadaverine, bacterial metabolism, lysine degradation pathway

## Abstract

There is a critical need to accurately diagnose, prevent, and treat biofilms in humans. The biofilm forming *P. aeruginosa* bacteria can cause acute and chronic infections, which are difficult to treat due to their ability to evade host defenses along with an inherent antibiotic-tolerance. Using an untargeted NMR-based metabolomics approach, we identified statistically significant differences in 52 metabolites between *P. aeruginosa* grown in the planktonic and lawn biofilm states. Among them, the metabolites of the cadaverine branch of the lysine degradation pathway were systematically decreased in biofilm. Exogenous supplementation of cadaverine caused significantly increased planktonic growth, decreased biofilm accumulation by 49% and led to altered biofilm morphology, converting to a pellicle biofilm at the air-liquid interface. Our findings show how metabolic pathway differences directly affect the growth mode in *P. aeruginosa* and could support interventional strategies to control biofilm formation.

## Introduction

*Pseudomonas aeruginosa* is a Gram-negative, opportunistic pathogen that exhibits resistance to many antibiotics, leading to acute and chronic infections in immunocompromised individuals (Hall-Stoodley et al., 2004). In 2017 the World Health Organization rated *P. aeruginosa* as a priority pathogen for research and development of new treatment strategies (Tacconelli et al., 2017). *P. aeruginosa’s* persistence is in part attributed to its ability to form biofilms, in which the cells are embedded in a gel-like matrix of self-produced extracellular polymeric substances (EPS), such as polysaccharides, proteins, and DNA. Biofilms have been shown to be up to 1,000 times more resistant to antibiotics than their planktonic counterparts and evade host immune responses (Lewis, 2001). *P. aeruginosa* biofilms are prevalent in respiratory illnesses such as cystic fibrosis, chronic wounds, and device related surgical site infections, among other conditions (Hall-Stoodley et al., 2004), yet there is a lack of effective strategies for diagnosis, prevention, and mitigation of biofilms.

Previous studies have begun to provide evidence for the vast physical and molecular differences between planktonic and biofilm growth modes, such as changes in motility (O'Toole and Kolter, 1998), quorum sensing (Wagner et al., 2007), and certain genomic (Whiteley et al., 2001), transcriptomic (Waite et al., 2005; Cornforth et al., 2018), and proteomic (Sauer et al., 2002) characteristics. Yet, these analyses identified many genes and proteins differentially expressed in biofilm that are not quorum-sensing related and some with no putative function (Whiteley et al., 2001; Sauer et al., 2002; Cornforth et al., 2018). This reflects that our understanding of signaling processes is still limited, and there likely exist unidentified regulons involved in biofilm formation. There is still much to uncover about the underlying biological mechanisms involved in the transition from planktonic to biofilm state along with a clear need for new experimental approaches and analysis methods (Toyofuku et al., 2016).

Complementary to other omics approaches, the comprehensive identification and quantification of small molecules involved in metabolic pathways (Sussulini, 2017) *via* metabolomics is particularly promising. Metabolites reflect the downstream changes of genes and enzymes, therefore, metabolomics will directly capture a snapshot of activity in the cells related to the growth mode (Sussulini, 2017). The power of nuclear magnetic resonance (NMR)-based metabolomics stems from its ability to highly reproducibly and non-destructively detect and quantify all abundant metabolites in a complex mixture in an untargeted manner (Gowda and Raftery, 2015; Markley et al., 2017).

Metabolomics has been used to gain insight into the molecular mechanism of biofilm formation in other bacterial pathogens (Yeom et al., 2013; Stipetic et al., 2016; Favre et al., 2018; Lu et al., 2019; Rieusset et al., 2020; Tang et al., 2021), but a comprehensive and quantitative analysis of metabolic changes involved in biofilm formation in *P. aeruginosa* is still missing (Gjersing et al., 2007; Zhang and Powers, 2012; Borgos et al., 2015). Detailed elucidation of major shifts in metabolic pathways in planktonic versus biofilm phenotypes has the potential to identify mechanisms of metabolic regulation, new targets for prevention and mitigation, and specific metabolic signatures for diagnosis of biofilms. Our study demonstrates NMR-based metabolomics as a viable approach to provide an unbiased, fully quantitative analysis to reveal metabolic pathway changes associated with the biofilm phenotype.

Here, we use 2D NMR spectroscopy to perform an untargeted metabolomics analysis of *P. aeruginosa* PAO1 grown planktonically and statically as a biofilm lawn for comparative analysis. Many metabolites identified show significant concentration changes between the two growth modes, including metabolites in the lysine degradation pathway (LDP). Using targeted metabolite supplementation with crystal violet (CV) staining and microscopy the differential role of this pathway could be unambiguously established suggesting new strategies toward biofilm monitoring and control.

## Materials and Methods

### Bacterial Strains, Growth Media, and Culturing Methods

*P. aeruginosa* strain PAO1 (Wilson et al., 2004) cultures were grown in lysogeny broth (LB) (Sigma Aldrich) shaking at 220 rpm at 37°C for 24 hours (hrs) to OD_600_ ≈ 1.0. Cultures were diluted in LB to OD_600_ = 0.1 then grown in LB culture or plated for metabolomics experiments. PAO1 was grown planktonically in 50 mL LB at 220 rpm at 37°C for 24 hrs (n = 9) and as a biofilm on LB plates (28.4 cm^2^) containing 1.5% (w/v) agar, statically, at 37°C in 5% CO_2_ for 48 hrs (n = 9). A red fluorescent PAO1 strain carrying a constitutively expressed Td-tomato producing plasmid pMQ400 (Locke et al., 2020), was cultured with 50 μg/mL gentamicin and utilized for visualization. PAO1 strain Xen41 (Xenogen Corp.), a luminescent strain carrying a constitutively expressed *lux*CDABE cassette, was utilized for visualization. CFU/mL (n = 6) and CFU/mL × cm^2^ (n = 4) were measured for planktonic and biofilm cultures, respectively, for metabolomics measurements by the microdilution plating technique (Pfeltz et al., 2001).

### Metabolomics Sample Preparation

Planktonic cultures were harvested by centrifugation at 4,300 × *g* for 20 min at 4°C. The pellet was washed by 1 mL phosphate-buffered saline (PBS) and transferred into a microcentrifuge tube (Eppendorf). Biofilm cultures were harvested by scraping with a sterile loop and transferring the biomass into two microcentrifuge tubes per sample due to the limited tube capacity. Samples were immediately re-suspended in 600 μL cold 1:1 methanol (Fisher)/double distilled H_2_O (ddH_2_O) for quenching. 300 μL of 1.4 mm stainless-steel beads (SSB14B) were added and cells were homogenized and lysed by a Bullet Blender (24 Gold BB24-AU by Next Advance) at a speed of 8 for 9 min at 4°C (Fuchs et al., 2016). An additional 500 μL 1:1 methanol/ddH_2_O was added and the sample was centrifuged at 14,000 × *g* for 10 min at 4°C to remove beads and solid debris. The supernatant was transferred to a 50 mL conical tube and 1:1:1 methanol/ddH_2_O/chloroform (Fisher) was added for a total volume of 24 mL (Bligh and Dyer, 1959; Leggett et al., 2019). The sample was vortexed and centrifuged at 4,300 × *g* for 20 min at 4°C for phase separation. The aqueous phase was collected, the methanol content was reduced using rotary evaporation, and lyophilized overnight. Before lyophilization 100 μL of each sample was saved for mass spectrometry (3.3% of total sample). For NMR measurements, the samples were re-suspended in 200 μL of NMR buffer (50 mM sodium phosphate buffer in D_2_O at pH 7.2 with 0.1 mM DSS (4,4-dimethyl-4-silapentane-1-sulfonic acid) for referencing) and centrifuged at 20,000 × *g* for 15 min at 4°C for removal of any residual protein content. The pellet was washed with 100 μL NMR buffer and the supernatants were combined and transferred to a 3 mm NMR tube with a Teflon cap and sealed with parafilm.

### NMR Experiments and Processing

NMR spectra were collected at 298 K on a Bruker AVANCE III HD 850 MHz solution-state spectrometer equipped with a cryogenically cooled TCI probe. 2D ^1^H-^1^H TOCSY spectra were collected (Bruker pulse program “dipsi2ggpphpr”) with 256 complex t_1_ and 2048 complex t_2_ points for a measurement time of 4 hrs. The spectral widths along the indirect and direct dimensions were 10,202.0 and 10,204.1 Hz and the number of scans per t_1_ increment was 14. 2D ^13^C-^1^H HSQC spectra (Bruker pulse program “hsqcetgpsisp2.2”) were collected with 512 complex t_1_ and 2048 complex t_2_ points for a measurement time of 16 hrs. The spectral widths along the indirect and direct dimensions were 34206.2 and 9375.0 Hz and the number of scans per t_1_ increment was 32. The transmitter frequency offset values were 75 ppm in the ^13^C dimension and 4.7 ppm in the ^1^H dimension for all experiments. NMR data was zero-filled four-fold in both dimensions, apodized using a cosine squared window function, Fourier-transformed, and phase-corrected using NMRPipe (Delaglio et al., 1995).

### NMR-Based Metabolomics Data Analysis

HSQC and TOCSY spectra were uploaded to the new COLMARq web server (**Supplementary Figure 2**) for peak picking, peak alignment, metabolite identification, metabolite quantification *via* Gaussian fitting, spectral normalization *via* a factor based on the average, median 30% peak volume ratios between an arbitrarily selected reference spectrum, and univariate statistical analysis between cohorts. Multivariate statistical analysis, hierarchical clustering analysis and heatmap visualization, and metabolite box plot analysis was performed *via* MetaboAnalyst (Xia et al., 2009). Metabolites were mapped to pathways *via* the KEGG PATHWAY database (Kanehisa et al., 2017).

### Mass Spectrometry Experiments

Lyophilized sample was dissolved in 1:1 acetonitrile (ACN)/ddH_2_O (v/v) with 0.1% formic acid and diluted 1×10^8^-fold for direct injection into a Q Exactive Plus Orbitrap mass spectrometer by ThermoFisher Scientific (resolving power of 280,000 and mass accuracy of <1 ppm). The instrument was internally calibrated with Thermo Scientific Pierce LTQ Velos ESI positive ion calibration solution and run in positive ion mode. The ionization method was electrospray ionization of 3.5 V. The mass range was set to 50-500 m/z. The flow rate was 3 μL/min with 0.9 scans/sec. Peak picking was done by PyOpenMS using a Gaussian width of 0.5. Peaks with amplitudes larger than one order of magnitude above the background were included as true peaks.

### Crystal Violet Staining Assays

Cadaverine (Sigma Aldrich) 5 or 10 mM stocks were prepared in LB and sterile filtered with a 0.2 μM filter. PAO1 overnight cultures were diluted to OD_600_ = 0.17 in LB with cadaverine in a concentration ranging from 0-3.30 mM. Cultures were plated in 96-well microtitre plates in at least triplicate, and incubated statically at 37°C in 5% CO_2_ for 24 hrs. Outer edge wells were filled with PBS to avoid “edge effects” due to evaporation (Sudhir K. Shukla, 2017). OD_600_ was measured to quantify planktonic growth. Liquid media was gently aspirated and wells were washed three times with 150 μL PBS. Adhered biofilm was stained with 125 μL of 0.1% crystal violet (CV) in 20% ethanol in ddH_2_O (v/v) for 30 min. CV was gently aspirated, wells were washed five times with 150 μL PBS, and CV was solubilized with 150 μL 33% glacial acetic acid in ddH_2_O (v/v) by shaking gently at 100 rpm at room temperature for 25 min. CV was quantified at OD_590_ to report biofilm accumulation. LB blanks were averaged and subtracted from readings. Control PAO1 wells were averaged and all measurements were normalized to control measurements per plate and reported as percent change from control.

### Confocal Laser Scanning Microscopy (CLSM)

PAO1 overnight cultures were diluted to OD_600_ = 0.17 in LB with 0 or 3.30 mM cadaverine in 35 × 10 mm confocal dishes and incubated statically at 37°C in 5% CO_2_ for 24 hrs. Liquid media was aspirated and adhered biofilm was stained with SYTO 9 for 10 min and washed with PBS. CLSM stitched images were collected (n=5) with a laser power of 4.5% under 10X magnification using an Olympus FluoView FV10i CLSM. Mean gray scale value and surface area coverage were quantified using Fiji (Schindelin et al., 2012).

### Culture Growth and Imaging By Photo, Dissecting Microscope, and IVIS

PAO1 Td-tomato or Xen41 cultures were grown similarly to CLSM cultures described above. iPhone 8 images were taken in a controlled well-lit environment. The air-liquid interface was imaged using an Amscope dissecting microscope with an MU500 camera. Xen41 light emission was detected with an IVIS Lumina II system (Caliper LifeSciences) as an indicator of biofilm activity.

### Statistical Analysis

All assays were performed in at least three independent replicates. Two-tailed unpaired Student’s t-tests were used for significant differences between groups. P-values below 0.05 were considered statistically significant. Metabolomics results were checked for multiple comparisons testing using the Benjamini-Hochberg false discovery rate (FDR) test (Benjamini and Hochberg, 1995). All error bars represent one standard error.

## Results

### Untargeted Metabolomics Analysis of *P. aeruginosa* in Planktonic and Biofilm States

**Figure 1:**
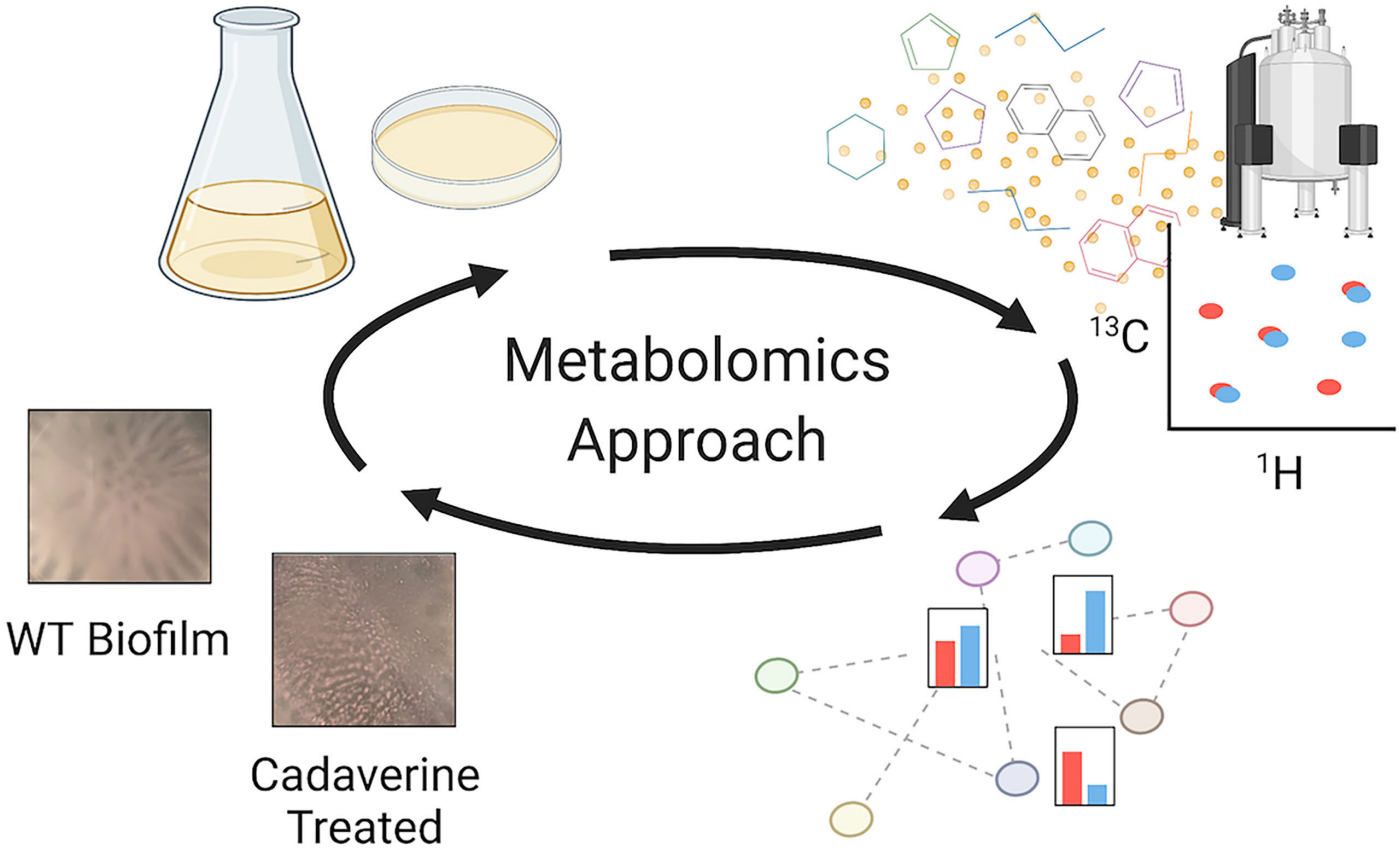
Metabolomics workflow to perform untargeted NMR-based metabolomics measurements, identify major metabolite changes, and test the role of the metabolites of interest. From the top left following the arrows: planktonic and biofilm PAO1 samples are prepared, and metabolites are extracted and measured by NMR; metabolites are then quantified and mapped to pathways for a biological interpretation; differentially quantified metabolites are tested to interpret their role in mode of growth. For example, there are significant decreases in the cadaverine pathway metabolites in biofilm compared to planktonic. When cadaverine is supplemented to the culture, biofilm accumulation is significantly reduced and morphology is altered. Created with Biorender.com.

**Figure 2:**
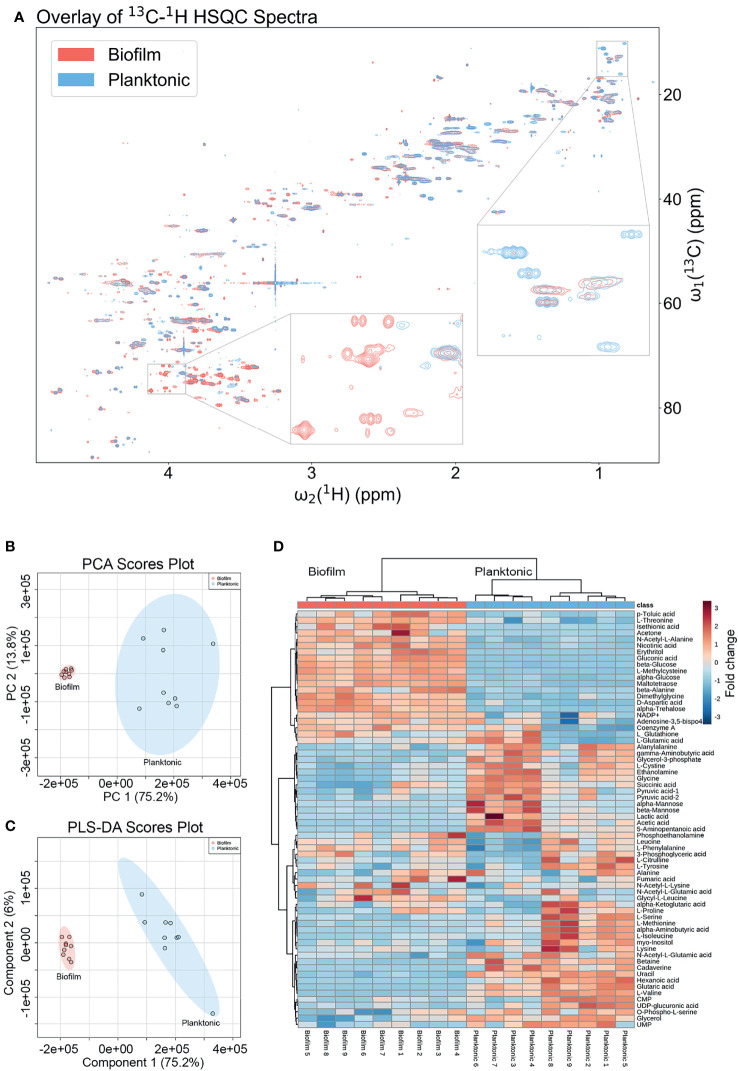
Metabolomics data analysis shows many unique metabolic differences between the biofilm and planktonic phenotypes. **(A)** Overlay of a representative region of the 2D ^13^C-^1^H HSQC spectra of a representative biofilm (red; bottom) and planktonic (blue; top) culture with select regions enlarged exemplifying peaks unique to each growth mode. Statistical analysis of metabolomics data distinguishes between biofilm and planktonic cohorts. Two-dimensional score plots for **(B)** principal component analysis (PCA) and **(C)** partial least squares discriminant analysis (PLS-DA) of biofilm (red) and planktonic (blue) sample cohorts (n=9) based on quantitation of identified metabolites show clustering of sample cohorts with no overlap of the ellipses (ellipses represent 95% confidence intervals), displaying good separation between and repeatability within cohorts. The heatmap **(D)** uses hierarchical clustering by Ward’s method and Euclidean distance to accurately cluster samples into their respective cohorts and the color scale shows metabolite fold changes between cohorts.

The metabolic differences between wild-type (WT) *P. aeruginosa* PAO1 grown in the free-floating planktonic and static lawn biofilm phenotypes were identified by NMR-based metabolomics and further investigated following the workflow in **Figure 1**. Lawn biofilms are known to generate large amounts of biomass and have been used to mimic bacterial growth on soft surfaces such as mucosal surfaces and tissue (Dusane et al., 2019; Hoiby et al., 2019). After growth both planktonic and biofilm samples yielded cultures of similar cell numbers for processing for NMR measurements (**Supplementary Figure 1**). All 2D NMR spectra were semi-automatically analyzed using our newly developed in-house COLMARq web server (**Supplementary Figure 2**) Representative planktonic and biofilm 2D ^13^C-^1^H HSQC spectra are shown as color-coded overlays in **Figure 2A**, with 1,302 distinct cross-peaks reflecting the rich content of detectable metabolites in these samples. A large number of peaks were only present either in the planktonic or the biofilm cultures signifying the presence of metabolites unique to each phenotype (**Figure 2A**). Of all cross-peaks, 436 were matched to known metabolites in the COLMAR database (Bingol et al., 2016). The remaining 866 HSQC peaks belong to “unknown” metabolites (**Supplementary Table 1**).

### Multivariate Analysis of Metabolomics Data by PCA and PLS-DA

Changes in quantitative metabolite concentrations were analyzed by both unsupervised and supervised multivariate statistical analysis methods, namely principal component analysis (PCA) and partial least squares discriminant analysis (PLS-DA) (**Figures 2B, C**). Both analyses organized the planktonic and biofilm samples into well-defined clusters and the groups did not show any overlap of the 95% confidence regions. The score plots of both analyses show a PC1 that comprises 75.2% of the variance in the entire data, which was dominated by the mean separation between the biofilm and planktonic sample cohorts. The PC2’s comprise 13.8% and 6.0% of the data variance for PCA and PLS-DA, respectively, which mostly reflect intra-cohort variability.

### Metabolites Differing Significantly Between Planktonic and Biofilm

All metabolites reported were identified with high confidence using the COLMARm method (Bingol et al., 2016) by querying ^13^C-^1^H HSQC spectra against the COLMAR database with subsequent confirmation by 2D ^1^H-^1^H TOCSY. A total of 66 unique metabolites, visualized in the heatmap in **Figure 2D**, were identified and quantified for comparison between planktonic and biofilm cohorts. Hierarchical clustering by Ward’s method and Euclidean distance show biofilm and planktonic samples were distinctly clustered into two groups (**Figure 2D**). Among the 66 distinct metabolites detected, the majority showed significant differences between the planktonic and biofilm phenotypes. 26 metabolites had a fold change greater than two, 52 metabolites had a statistically significant difference with *p*<0.05, 44 metabolites with *p*<0.01, and 14 metabolites with *p*<1.00×10^-7^. An additional 14 metabolites showed no significant change (**Supplementary Table 2**) with many of them likely to be serving as housekeeping metabolites, including 11 amino acids and their conjugates or involvement in central metabolism, such as glycolysis and the TCA cycle.

**Figure 3:**
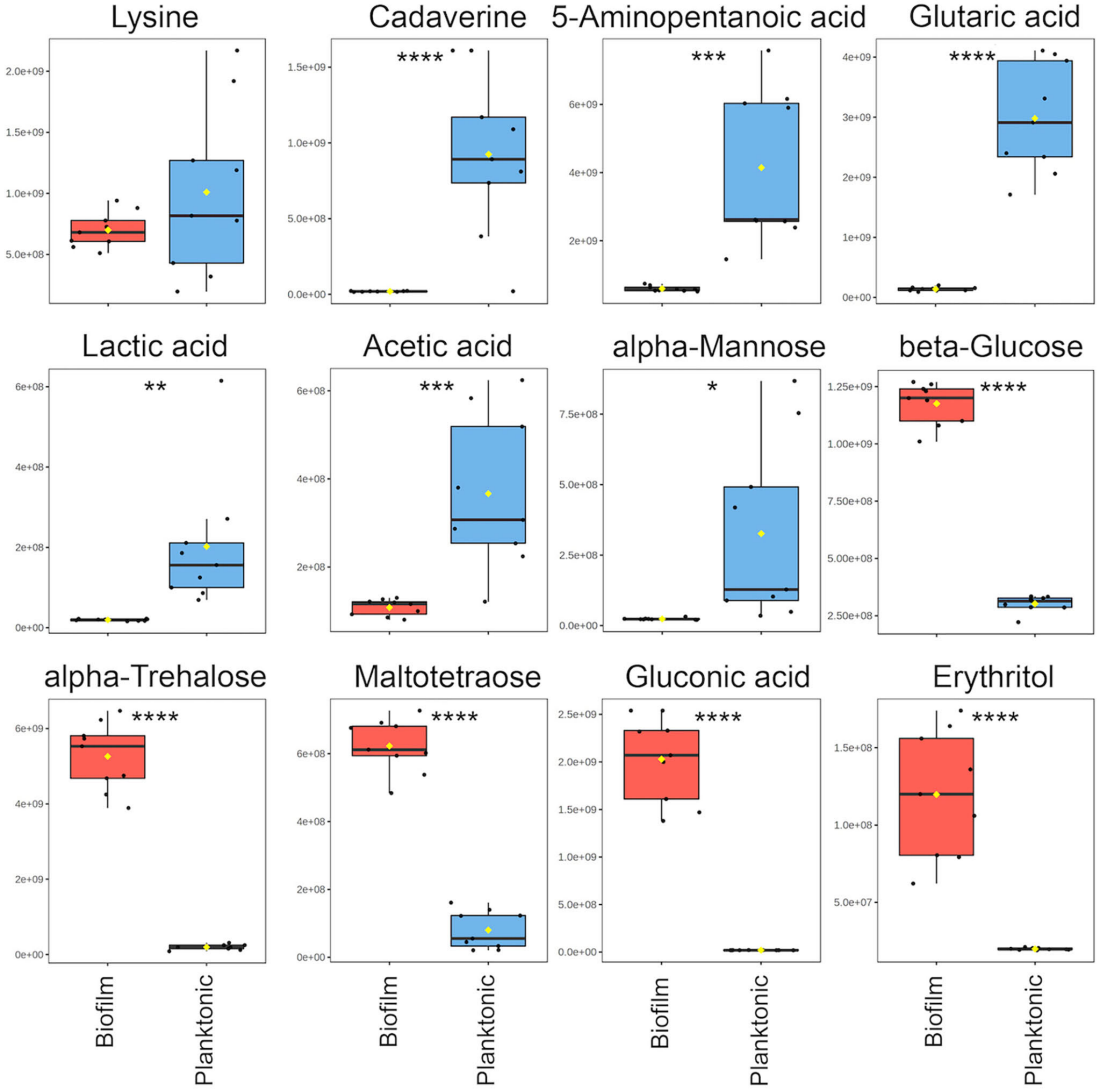
Notable metabolite differences between planktonic and biofilm include intermediates of the cadaverine branch of the lysine degradation pathway (LDP), weak organic acids, and carbohydrate-related metabolites. Box plots represent a metabolite fold change analysis between biofilm (red) and planktonic (blue) cohorts (n=9). The black circles represent independent sample values, boxes represent upper and lower quartiles, black bars represent median, yellow diamonds (♦) represent mean value, whiskers represent minimum and maximum values, and asterisks denote significance (*p < 0.05, **p < 0.01, ***p < 0.001, ****p < 0.0001 by unpaired, two-tailed *t*-test).

For the comparative quantitative analysis, we treat the planktonic metabolite quantities as reference and report relative changes in the biofilm. Metabolites with the most notable differences, having a fold change greater than two and *p*<0.05, include 14 metabolites in biofilm that were significantly increased and 11 metabolites that were significantly decreased (**Supplementary Table 2**). Selected metabolites with interpreted potential roles are shown in **Figure 3**. A majority of metabolites whose abundance increased in biofilm were carbohydrate-related, such as mono- and disaccharides, sugar acids and alcohols, which increased from four to 102-fold. The only identified carbohydrate with the opposite trend was mannose, with about a six-fold decrease in biofilm. This is consistent with previous studies, which suggested that mannose acts as a competitive inhibitor of bacterial adherence by binding adhesion proteins or lectins in both *E. coli* and *P. aeruginosa* (Hauck et al., 2013; Scaglione et al., 2021). Weak organic acids (WOA) such as lactic and acetic acid were significantly decreased in biofilm about 10-fold and three-fold, respectively (**Figure 3**). A recent study showed that addition of lactic and acetic acid to culture in *E. coli* reduced production of extracellular polymeric substances, inhibited quorum sensing, and reduced biofilm formation (Amrutha et al., 2017). Therefore, lactic and acetic acid may also play a role in establishing growth mode in *P. aeruginosa*, favoring the planktonic phenotype.

In addition to the metabolites identified by querying the HSQC spectrum against known metabolites, 66.5% of the HSQC peaks belong to compounds that could not be matched to metabolites in the COLMAR database. Of these “unknown” peaks, 493 show statistically significant differences between planktonic and biofilm cohorts (**Supplementary Table 1**). Based on the average number of identified peaks per known metabolite, these “unknown” signals could belong to an estimated 75 additional metabolites differing significantly in quantity between phenotypes. Identification of the unknown metabolites and linking them to new proteins and biochemical pathways is an opportunity to advance our understanding of other biochemical changes that accompany the phenotypic changes and may offer new targets for biofilm control.

### Identification of the Lysine Degradation Pathway for Its Possible Role in *P. aeruginosa* Growth Mode

**Figure 4:**
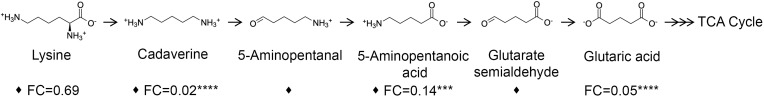
Metabolites of the cadaverine branch of the lysine degradation pathway are significantly down-regulated in biofilm compared to planktonic cultures. Lysine is degraded to supplement the TCA cycle to increase cellular respiration. Metabolites identified and quantified by NMR show a fold change (FC) (biofilm/planktonic) (n=9) and asterisks denote significance (***p < 0.001, ****p < 0.0001 by unpaired, two-tailed *t*-test). Metabolites with at least one common adduct detected by mass spectrometry by direct injection in positive ion mode with a mass error of <0.77 ppm are denoted by a black diamond (♦).

Three metabolites including cadaverine (biofilm/planktonic = 0.02; p = 8.74 × 10^-5^), 5-aminopentanoic acid (biofilm/planktonic = 0.14; p = 2.17 × 10^-4^), and glutaric acid (biofilm/planktonic = 0.05; p = 7.03 × 10^-8^) (**Figures 3**, **4**) were found to be significantly decreased in biofilm and could be mapped on the cadaverine branch of the LDP (**Figure 4**) using the KEGG PATHWAY database (Fothergill and Guest, 1977; Indurthi et al., 2016; Kanehisa et al., 2017). The LDP is an important link in central metabolism as lysine is typically taken up from the growth media *via* transport channels and degraded to glutaric acid, which enters the tricarboxylic acid (TCA) cycle (Knorr et al., 2018). The majority of metabolites of the cadaverine branch of the LDP were detected and quantified by our NMR-based metabolomics analysis, namely lysine, cadaverine, 5-aminopentanoic acid, and glutaric acid (**Figure 4**). Since other intermediates of the LDP, namely 5-aminopentanal and glutarate semialdehyde, were not present in the COLMAR database, their presence based on NMR spectra alone is unknown. However, based on mass spectrometry they are likely to be present as at least one common adduct of each of these metabolites was detected within less than 0.77 ppm of their expected mass. All metabolites in the cadaverine pathway were detected by mass spectrometry for additional confirmation except for glutaric acid, which contains two carboxylic acid groups that are negatively charged and therefore may not be detectable in positive ion mode used here (**Supplementary Table 3**). While many metabolites of the cadaverine branch of the LDP were significantly different, lysine did not show a significant fold change (biofilm/planktonic = 0.69; p = 0.21) (**Figures 3**, **4**).

### Exogenous Supplementation of Cadaverine Increases Planktonic Growth and Inhibits Biofilm Accumulation

**Figure 5:**
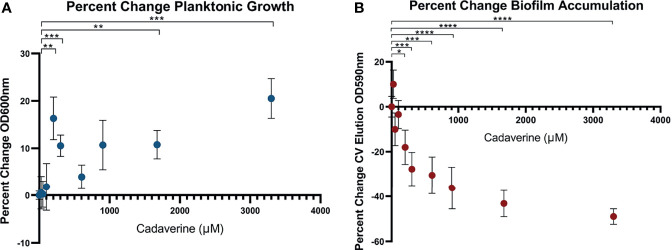
Microtitre plate assays show cadaverine supplementation significantly increases planktonic growth and decreases biofilm accumulation post inoculation. Planktonic growth **(A)** and biofilm accumulation **(B)** are measured by OD_600_ and crystal violet staining elution at OD_590_, respectively, after supplementation of 0-3.30mM cadaverine to the growth media for 24 hrs (n=18). Values are normalized to the control wells (no cadaverine) in each plate and reported as a percent change from control and asterisks denote significance (*p < 0.05, **p < 0.01, ***p < 0.001, ****p < 0.0001 by unpaired, two-tailed *t*-test). **(A)** Planktonic growth significantly increases at several cadaverine concentrations, maximally by 21%. **(B)** Biofilm accumulation significantly decreases in a cadaverine concentration dependent manner, maximally by 49%.

Cadaverine belongs to a class of compounds known as polyamines, which have been reported to perform multiple roles in bacteria with links to cell growth, proliferation, bacterial carcinogenesis, escape from phagolysosomes, bacteriocin production, natural product synthesis, toxin activity, protection from oxidative and acidic stress, and electrostatic interactions (Shah and Swlatlo, 2008; Michael, 2016). Polyamines, such as spermidine and putrescine, have been shown to play a direct role in biofilm formation in *Vibrio cholera*, *Yersinia pestis*, *E. coli*, *Bacillus subtilis*, and *Neisseria gonorrheoae* (Karatan and Michael, 2013), yet it remains unknown whether there is a common function of polyamines in all biofilm-forming bacteria. Of the LDP intermediates, we find that cadaverine shows the largest difference between phenotypes and therefore may play a role in establishing growth mode. It is known that metabolites of a targeted pathway can be supplemented to trigger rapid changes in enzyme activity leading to reprogrammed metabolic activity (Wegner et al., 2015). To test this possibility for the cadaverine branch of the LDP, we supplemented cadaverine to the growth media in a concentration range of 0 - 3.30 mM and concurrently measured planktonic growth by OD_600_ and biofilm accumulation by crystal violet (CV) staining elution at OD_590_ after 24 hrs. With the addition of cadaverine, planktonic growth increased significantly whereas biofilm accumulation decreased significantly (**Figures 5A, B**). Moreover, addition of cadaverine was not bactericidal as the OD_600_ increased at most concentrations. Planktonic growth increases in a somewhat cadaverine-concentration dependent manner, however not all concentrations altered growth significantly, which is likely due to the variability in the planktonic samples. Planktonic growth increased maximally by 20.5 ± 4.2% with 3.30 mM cadaverine (**Figure 5A**). This contrasts the biofilm response to exogenous cadaverine: at low cadaverine concentration (25 μM) biofilm accumulation increased marginally before systematically decreasing with 200 μM to 3.30 mM cadaverine, leveling off at a 49.0 ± 3.5% decrease at the highest cadaverine concentration (**Figure 5B**). These results suggest that exogenous supplementation of cadaverine stimulates planktonic growth and inhibits biofilm accumulation post inoculation.

Because of its basic nature, cadaverine supplementation increased the pH of the media by up to 0.8 pH units (**Supplementary Figure 3A**) with the pH remaining in the normal growth range for *P. aeruginosa* (Klein et al., 2009). This small pH change by itself did not cause increased planktonic growth or reduced biofilm accumulation, as increasing the pH by addition of sodium hydroxide in lieu of cadaverine caused no systematic significant change in planktonic growth or biofilm accumulation (**Supplementary Figure 3B**).

### Confirmation of Cadaverine Inhibition of Biofilm Accumulation by Confocal Laser Scanning Microscopy

**Figure 6:**
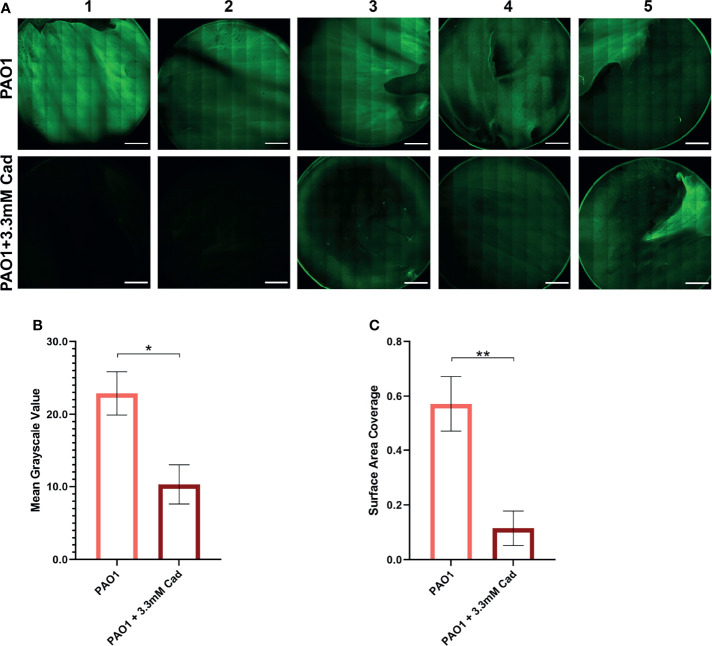
Stitched confocal microscopy images show a significant reduction in biofilm accumulation with cadaverine by image quantification. **(A)** 3.30mM cadaverine (cad) is supplemented to the PAO1 culture in a 35 by 10mm dish, grown 24 hrs, stained with SYTO 9, and stitched confocal images (n=5; paired to control cultures) are taken at laser power 4.5%. Scale bars in the lower right corner of images represent 0.2 cm. Images are quantified in Fiji by **(B)** mean grayscale value and **(C)** surface area coverage with the auto threshold set to 13 and asterisks denote significance (*p < 0.05, **p < 0.01 by unpaired, two-tailed *t*-test). Mean grayscale value and surface area coverage show 55% and 80% reduction in biofilm accumulation, respectively, with supplementation of cadaverine.

To independently confirm the CV elution results by an alternative method, biofilm accumulation was measured by CLSM. 3.30 mM cadaverine was supplemented to the growth media in a confocal dish. At 24 hrs, samples were stained with SYTO 9 and stitched confocal images were collected and quantified by Fiji (Schindelin et al., 2012) (**Figure 6A**). Mean grayscale value (**Figure 6B**) showed a significant reduction in biofilm accumulation of 54.5 ± 26.0% and surface area coverage (**Figure 6C**) showed a significant reduction in biofilm accumulation by 79.8 ± 55.1%. Both visual inspection and quantification of the biofilm images show variation among replicates, likely partially due to the inherently heterogeneous nature of biofilms (Stewart and Franklin, 2008). Despite the heterogeneity, the average effect shows significant reduction in biofilm accumulation with cadaverine supplementation.

### Exogenous Supplementation of Cadaverine Inhibits Biofilm Accumulation in the Presence of Pre-Formed Biofilm

To investigate whether cadaverine could also inhibit biofilm accumulation in the presence of pre-formed biofilm, cultures were grown for 24 hrs then supplemented with cadaverine and grown an additional 24 hrs for OD_600_ and CV staining elution. Planktonic growth was significantly increased by 5.8 ± 1.8% (**Supplementary Figure 4A**) and biofilm accumulation was significantly decreased by 39.8 ± 2.5% (**Supplementary Figure 4B**). These results are consistent with the previous assay, demonstrating that addition of cadaverine stimulates planktonic growth and inhibits biofilm accumulation, even in the presence of pre-formed biofilm.

### Exogenous Supplementation of Cadaverine Causes a Macroscopic Alteration in Biofilm Morphology

The aforementioned assays measured biofilm accumulation of surface-attached biofilm after aspiration, washing, and staining. We also report a macroscopic alteration of biofilm morphology in standing liquid culture dishes grown for 24 hrs with cadaverine. The *P. aeruginosa* Td-tomato strain, utilized for visualization, supplemented with cadaverine showed a significant increase in planktonic growth by OD_600_ (**Supplementary Figure 5A**) and significant reduction in biofilm accumulation by CV staining elution (**Supplementary Figure 5B**), similarly to WT PAO1.

**Figure 7:**
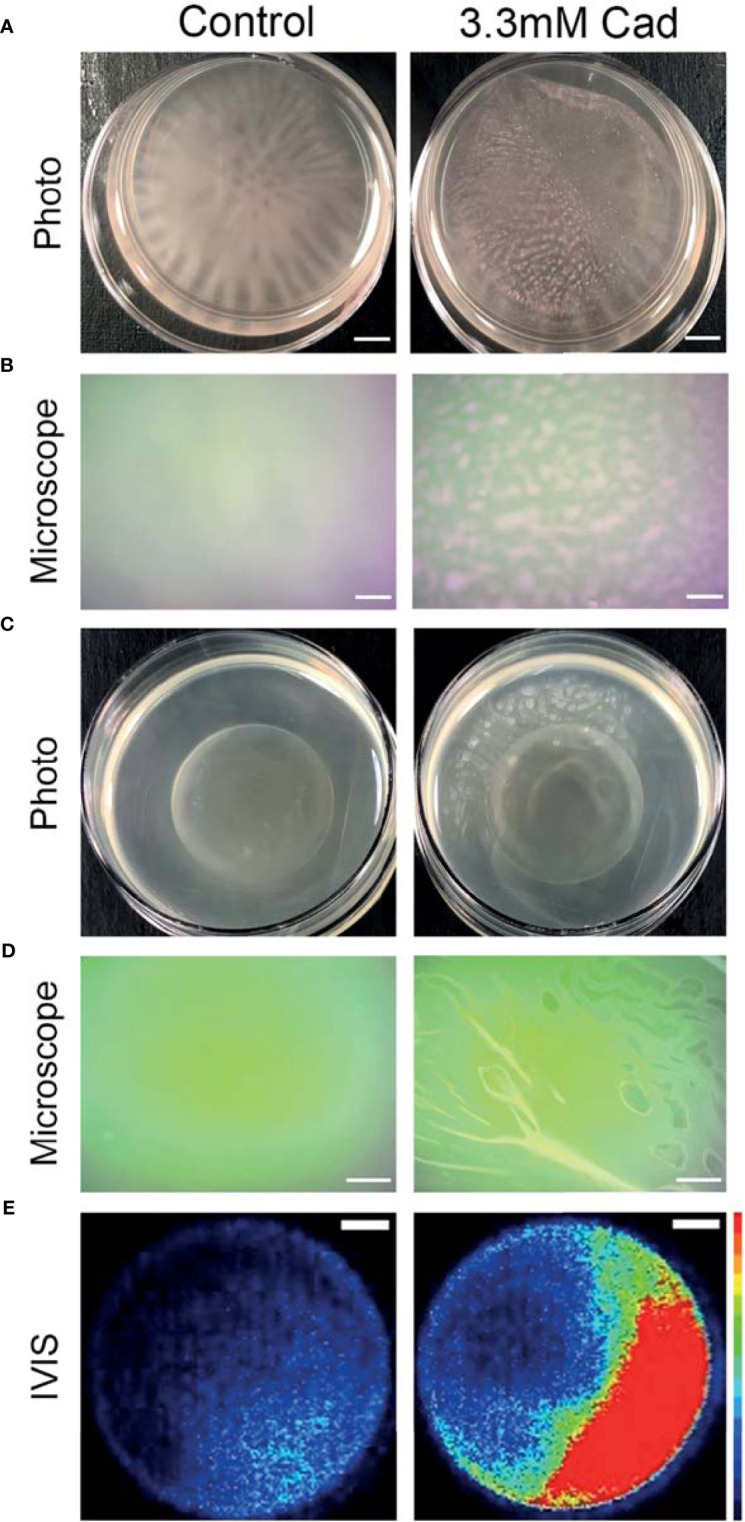
Cadaverine supplementation causes altered biofilm morphology to pellicle biofilm at the air-liquid interface that is metabolically active. Representative iPhone8 photos (scale bar = 2.9mm) **(A)** and dissecting microscope images of culture air-liquid interfaces (scale bar = 0.14mm) **(B)** are taken of PAO1 Td-tomato supplemented with 3.30mM cadaverine (cad) showing pellicle biofilm. Representative iPhone8 photos (scale bar = 3.6mm) **(C)**, dissecting microscope images of culture air-liquid interfaces (scale bar = 0.18mm) **(D)**, and IVIS images of air-liquid interfaces (scale bar = 3.8mm) with red being the most metabolically active (scale on right of images) **(E)** are shown of PAO1 Xen41 supplemented with 3.30mM cadaverine. Cadaverine supplementation leads to biofilm formation at the air-liquid interface compared to the control, where biofilm is localized to the bottom of the dish.

Representative macroscopic images of cultures in **Figure 7A** revealed altered biofilm morphology in the presence of supplemented cadaverine. In control cultures the biofilm appears in a web-like structure that is well attached and localized at the bottom of the dish. With 3.30 mM cadaverine, the biofilm appears more aggregate-like, in a pellicle, which consists of many small areas of biofilm accumulation toward the air-liquid interface (**Figure 7A**). The air-liquid interface of the cultures was imaged using an AmScope MU500 camera with an AmScope dissecting microscope, showing a microscopic image of the areas of pellicle biofilm appearing at the air-liquid interface with the addition of cadaverine (**Figure 7B**). In the control, no biofilm growth appears at the air-liquid interface.

Macroscopic photos, dissecting microscope images, and IVIS images in **Figures 7C–E** were also collected with the constitutive bioluminescent PAO1 derivative Xen41. Representative photos (**Figure 7C**) and dissecting microscope images (**Figure 7D**) of the air-liquid interface similarly showed pellicle biofilm with cadaverine supplementation. To determine the metabolic state of the bacteria within the pellicle and surrounding supernatant, we utilized an IVIS imaging system, which captures light from bioluminescence that is proportional to the cell numbers and metabolic activity. IVIS images of cultures with cadaverine supplemented show that the pellicle biofilm emits more light, which is proportional to the accumulation of more cells in biofilm at the air-liquid interface (**Figure 7E**). The light production is localized to the areas of pellicle, indicating that they are metabolically active (**Figure 7E** and **Supplementary Figure 6**). In contrast, biofilm attached at the bottom of the dish in the control cultures emits low levels of luminescence, likely resulting from reduced oxygen and a limited dynamic range with the strong signal collected at the air-liquid interface of the test cultures. Therefore, along with significantly less total biofilm accumulation, cadaverine supplementation stimulates the altered macroscopic morphology to a pellicle biofilm. We also found the cadaverine-induced pellicle biofilm was more easily aspirated than the more strongly attached, web-like control biofilm (**Figure 7**). This likely contributes to the reduced surface-attached biofilm accumulation measured *via* the CV assay and microscopy (**Figures 5**, **6**).

## Discussion

Our untargeted NMR-based metabolomics approach enabled us to uncover specific metabolites and pathways involved in regulation of growth mode and biofilm formation. Of the significant metabolite changes identified by our new COLMARq web server, the cadaverine branch of the LDP was the pathway that showed the most significant differences between planktonic and biofilm phenotypes, with significant reduction in the biofilm phenotype. Exogenous supplementation of cadaverine to cultures significantly stimulated planktonic growth and inhibited biofilm accumulation (**Figure 5**). This suggests exogenous supplementation of cadaverine may reprogram cellular metabolism to maintain a more planktonic-like metabolic state leading to reduced biofilm formation. To our knowledge, this is the first association of the cadaverine pathway to biofilm accumulation in *P. aeruginosa*. Other polyamines have been shown to play varied roles in biofilm formation in other pathogens (Rojo, 2010), indicating that the role of each polyamine is specific, requiring a separate mechanistic investigation for each system.

The observed stimulation of planktonic growth by cadaverine reported here may be related to previous findings of possible roles of cadaverine, including combatting cellular stress (Miller-Fleming et al., 2015; Soksawatmaekhin et al., 2004), increase of cell viability in the stationary phase (Sakamoto et al., 2012), stimulation of protein synthesis (Igarashi and Kashiwagi, 2018), and increase of cellular respiration and growth (Nie et al., 2017; Knorr et al., 2018), thereby contributing to the favoring of the planktonic phenotype. Polyamines in *E. coli* have been shown to form complexes with RNA, stimulate assembly of the ribosome, and increase general protein synthesis about two-fold (Igarashi and Kashiwagi, 2018), which may contribute to increased growth. The cadaverine pathway also increases cellular respiration by supplementing the TCA cycle (Nie et al., 2017; Knorr et al., 2018). Metabolic intermediates such as cadaverine and glutaric acid serve as better carbon and nitrogen sources than lysine itself (Fothergill and Guest, 1977; Indurthi et al., 2016). Since nutrient restriction has been associated with stimulating biofilm formation (Zhang et al., 2012), increased metabolic activity in *P. aeruginosa* may generally favor the planktonic phenotype.

Alternatively, inhibition of biofilm accumulation by cadaverine may be related to its ability to alter adhesion protein expression (Torres et al., 2005). Restoration of lysine decarboxylase after inhibition to produce cadaverine in *E. coli* led to reduced production of intimin, an adhesion protein (Torres et al., 2005). Therefore, cadaverine may act on biofilm matrix components contributing to more weakly attached biofilms and reduced biofilm accumulation.

Another proposed mechanism of the regulation of biofilm formation in *P. aeruginosa* is modulation of the second messenger bis-(3’-5’)-cyclic dimeric guanosine monophosphate (c-di-GMP) (Romling et al., 2013; Randall et al., 2021). In most cases, the mechanism that leads to alterations in c-di-GMP levels is unknown (Barrientos-Moreno et al., 2020). However, a most recent study shows that the polyamine putrescine and its metabolic precursor L-arginine increase biofilm formation in *P. aeruginosa*, at least in part through increasing c-di-GMP levels (Liu et al., 2021). Cadaverine production through lysine catabolism is coupled with arginine metabolism *via* the arginine-responsive regulator (Chou et al., 2010). This association could suggest that cadaverine regulates biofilm formation in part through affecting c-di-GMP levels. Measuring metabolic pathway changes and c-di-GMP after exogenous cadaverine supplementation is an interesting future direction to inform further on metabolic mechanisms underlying biofilm regulation.

Our findings highlight the potential of NMR-based metabolomics as a viable tool for diagnosis and identification of new targets for prevention and control of *P. aeruginosa* infection and biofilm. Detection of certain types of infections, such as periprosthetic joint infection, is difficult and requires time-consuming culturing methods, making early and pathogen-specific intervention unfeasible in many cases and there are no clinical biomarkers for the presence of biofilms (Glaudemans et al., 2019; Wasterlain et al., 2020). As a highly reproducible and quantitative method, NMR spectroscopy has the potential to identify metabolite biomarkers or fingerprints of infection in bodily fluids such as serum and synovial fluid (Hugle et al., 2012; Ammons et al., 2014; Palama et al., 2016). With 782 HSQC peaks identified that manifest significant concentration fold changes (up to 100-fold) between *P. aeruginosa* planktonic and biofilm, unique metabolites or metabolomic signatures, if detectable *in vivo*, could be used as culture-free diagnostic markers. These markers have the potential to enable rapid identification of bacterial growth mode and to aid in deciding the optimal treatment. In addition, combining biofilm-controlling compounds with antibiotics has been identified as an effective strategy to control biofilm infections (Estrela and Abraham, 2010). We found supplementation of cadaverine did not have a bactericidal or bacteriostatic effect while reducing biofilm accumulation post inoculation and in the presence of pre-formed biofilm, indicating that it could act to reduce biofilm formation and potentially increase susceptibility to antibiotics. Previous studies have shown cadaverine enhances the effectiveness of many β-lactams against *P. aeruginosa* (Kwon and Lu, 2006; Manuel et al., 2010). Cadaverine is a natural metabolite found in all living organisms (Miller-Fleming et al., 2015) and can stem from microbiota or have endogenous origin (Amin et al., 2021). Cadaverine supplementation shows low acute oral toxicity at 2,000 mg/kg body weight in rats (Til et al., 1997), and negligible cytotoxicity up to 70 mM in HT29 intestinal cells (del Rio et al., 2019). Therefore, adding cadaverine to the *P. aeruginosa* infection prevention or treatment course is a potentially viable new strategy that warrants further investigation.

Our quantitative untargeted metabolomics approach can be directly applied to the characterization of biofilm versus planktonic phenotypes of *P. aeruginosa* and other biofilm-forming bacteria in growth media and environments other than those studied here. Our cultures were grown in LB media that contains a mixture of carbon sources including amino acids and glucose among other nutrients. *P. aeruginosa* utilizes a carbon catabolite repression system to select preferred carbon sources and optimize growth in various environments (Karatan and Michael, 2013). *P. aeruginosa* prefers organic acids or amino acids over standard nutrients like glucose. With preferred nutrients available, we detected alteration of the LDP, an amino acid pathway, to modulate growth between planktonic and biofilm phenotypes. Utilizing other growth conditions will provide more insight about the generality of the role of the lysine degradation pathway in *P. aeruginosa* biofilm formation or even may lead to the identification of other endogenous metabolites that can be used to modulate biofilm growth. In addition, utilizing clinical isolates and mimicking specific environments, for example artificial sputum in the case of cystic fibrosis lung infections or synovial fluid in the case of periprosthetic knee or hip joint infections, is expected to provide important new information about metabolic pathways associated with biofilm growth in specific conditions.

In conclusion, we have identified and quantified many specific metabolic differences between *P. aeruginosa* planktonic and biofilm phenotypes and discovered that the cadaverine pathway is linked to the establishment of the growth mode. Exogenous cadaverine supplementation to cultures led to stimulated planktonic growth, inhibited biofilm accumulation by up to 49%, and induced macroscopic pellicle biofilm structure. Our findings identify cadaverine and the LDP as a potential target for prevention and mitigation of *P. aeruginosa* biofilm infections. Additional studies of the effect of cadaverine metabolism in *P. aeruginosa* clinical isolates and the virulence and persistence of the induced pellicle biofilm *in vivo* are needed. Beyond cadaverine alone, treatment with a mixture of LDP pathway intermediates or other endogenous metabolites that are decreased in the biofilm phenotype could further increase effectiveness in modulating growth mode. Further advances in our understanding of the precise role of metabolic regulation in biofilm formation open new possibilities to the modulation of growth mode in *P. aeruginosa*. The metabolomics approach used here should be applicable to other biofilm-forming bacteria to deepen our understanding of the existence and role of metabolic pathways in these pathogens.

## Data Availability Statement

The original contributions presented in the study are included in the article/**Supplementary Material**. Further inquiries can be directed to the corresponding authors.

## Author Contributions

Conceptualization and design: AL, LB-L, RB, and PS. Investigation: AL, DS, AS, and NR. Data analysis: AL and DL. Figures: AL and NR. Writing of the original draft: AL, RB, and PS. All authors read and approved the final version of the manuscript.

## Funding

This work was supported by the National Institutes of Health [grants R01GM124436 (to PS), R01GM066041, and R35GM139482 (to RB)] and by a pilot grant from the Department of Microbial Infection and Immunity in the College of Medicine at OSU. 

## Conflict of Interest

The authors declare that the research was conducted in the absence of any commercial or financial relationships that could be construed as a potential conflict of interest.

## Publisher’s Note

All claims expressed in this article are solely those of the authors and do not necessarily represent those of their affiliated organizations, or those of the publisher, the editors and the reviewers. Any product that may be evaluated in this article, or claim that may be made by its manufacturer, is not guaranteed or endorsed by the publisher.
