# Supplementary material for: Cadaverine Is a Switch in the Lysine Degradation Pathway in Pseudomonas aeruginosa Biofilm Identified by Untargeted Metabolomics
Source: Front Cell Infect Microbiol. 2022 Feb 14;12:833269. doi: 10.3389/fcimb.2022.833269 (PMC8884266; doi:10.3389/fcimb.2022.833269)
Supplement: Supplementary file 1 [file DataSheet_1.zip › Supplementary Material Presentation/SuppTable3.docx]

| Metabolite | Formula | Monoisotopic mass (g/mol) | Adducts | Intensity | Mass error (ppm) |
| --- | --- | --- | --- | --- | --- |
| Lysine | C_6_H_14_N_2_O_2_ | 146.105528 | +1 H’ | 1.46E+05 | 0.51 |
|  |  |  | +1 ACNH’ | 8.61E+04 | 1.04 |
|  |  |  | +2 H’ | 1.20E+07 | 0.61 |
| Cadaverine | C_5_H_14_N_2_ | 102.115698 | +1 H’ | 1.09E+06 | 1.15 |
|  |  |  | +1 ACNH’ | 1.15E+06 | 0.76 |
| 5-Aminopentanal | C_5_H_11_NO | 101.084064 | +1 H’ | 1.82E+06 | 0.94 |
|  |  |  | +1 ACNH’ | 6.19E+05 | 0.61 |
|  |  |  | +1 NH4’ | 7.48E+04 | 0.79 |
| 5-Aminopentanoic acid | C_5_H_11_NO_2_ | 117.0789786 | +1 H’ | 5.61E+05 | 1.24 |
|  |  |  | +1 ACNH’ | 5.49E+05 | 0.42 |
| Glutarate semialdehyde | C_5_H_8_O_3_ | 116.0473441 | +1 H’ | 1.48E+04 | 1.03 |
|  |  |  | +1 NH4’ | 4.46E+04 | 0.71 |
