# Supplementary figures and images for: Cadaverine Is a Switch in the Lysine Degradation Pathway in Pseudomonas aeruginosa Biofilm Identified by Untargeted Metabolomics
Source: Front Cell Infect Microbiol. 2022 Feb 14;12:833269. doi: 10.3389/fcimb.2022.833269 (PMC8884266; doi:10.3389/fcimb.2022.833269)

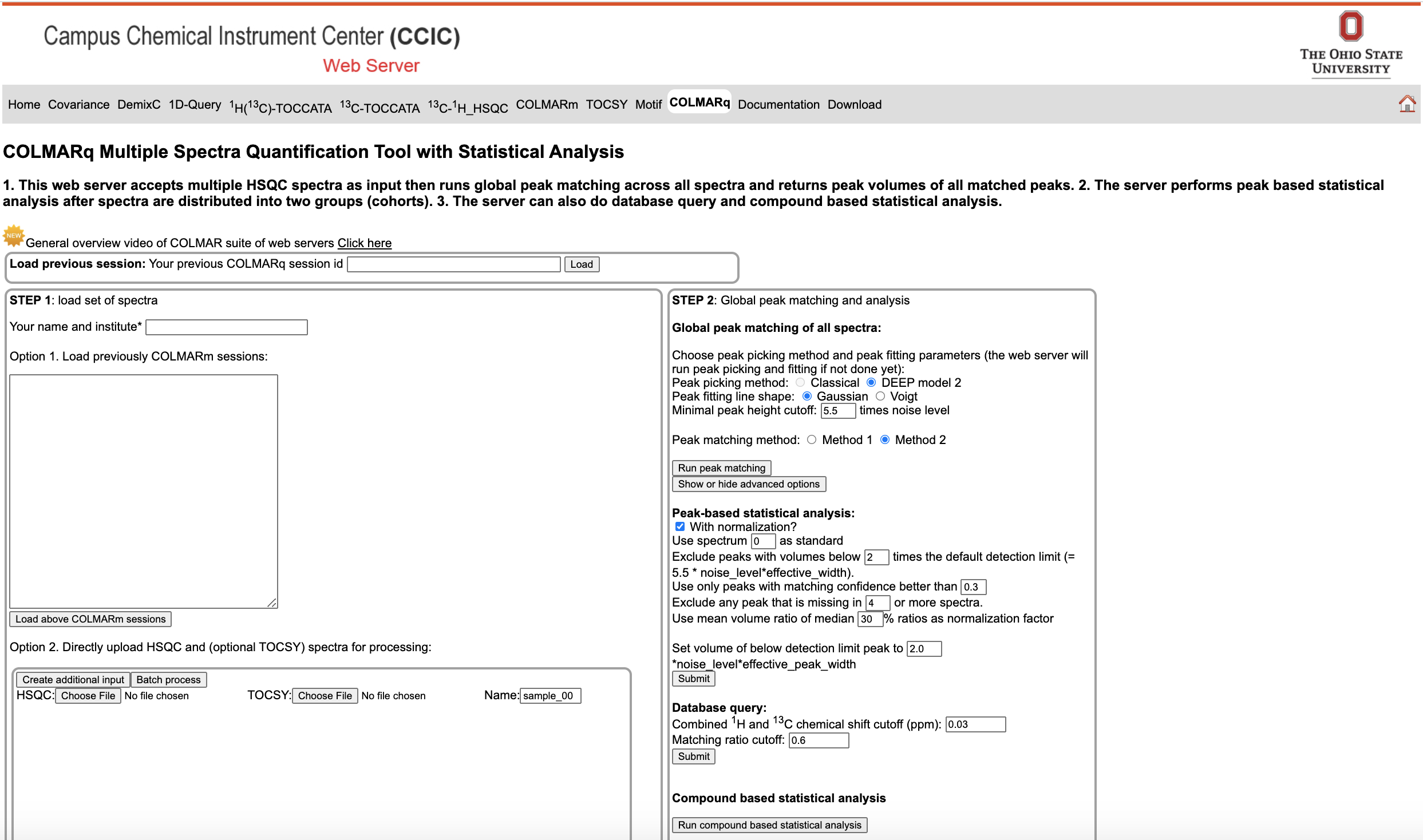

Supplement: Supplementary file 1 [file DataSheet_1.zip › Supplementary Material Presentation/SuppFig2.png]

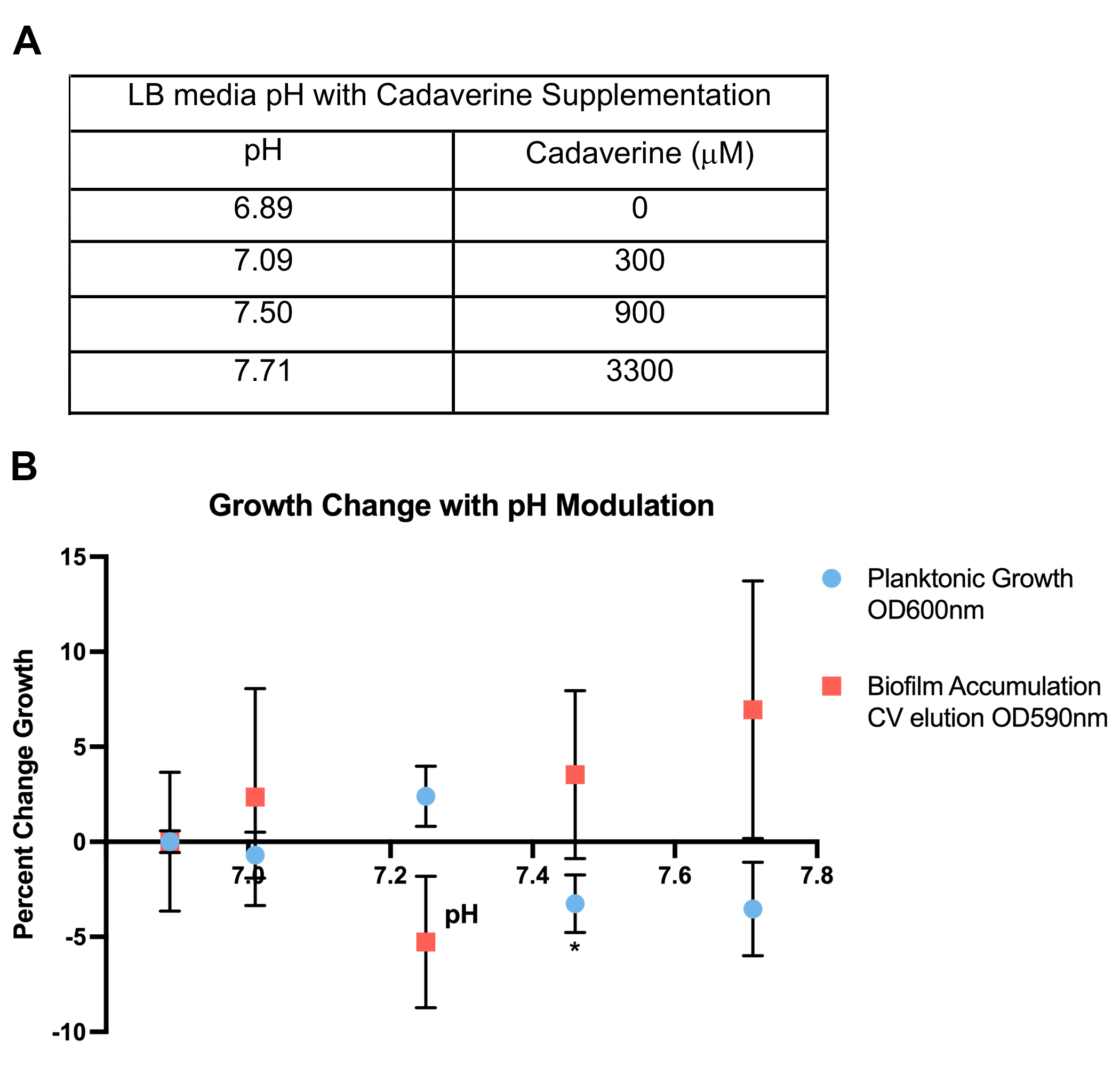

Supplement: Supplementary file 1 [file DataSheet_1.zip › Supplementary Material Presentation/SuppFig3.jpg]

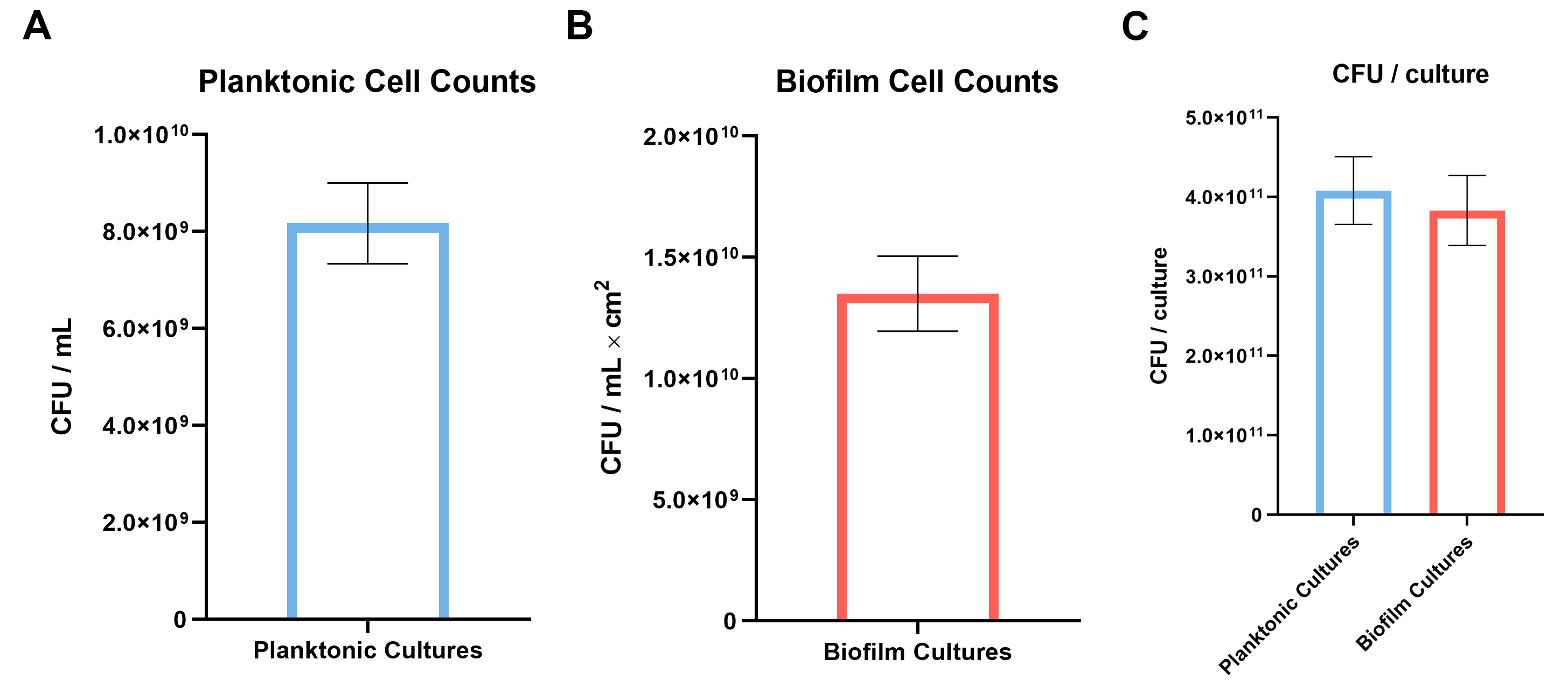

Supplement: Supplementary file 1 [file DataSheet_1.zip › Supplementary Material Presentation/SuppFig1.jpg]

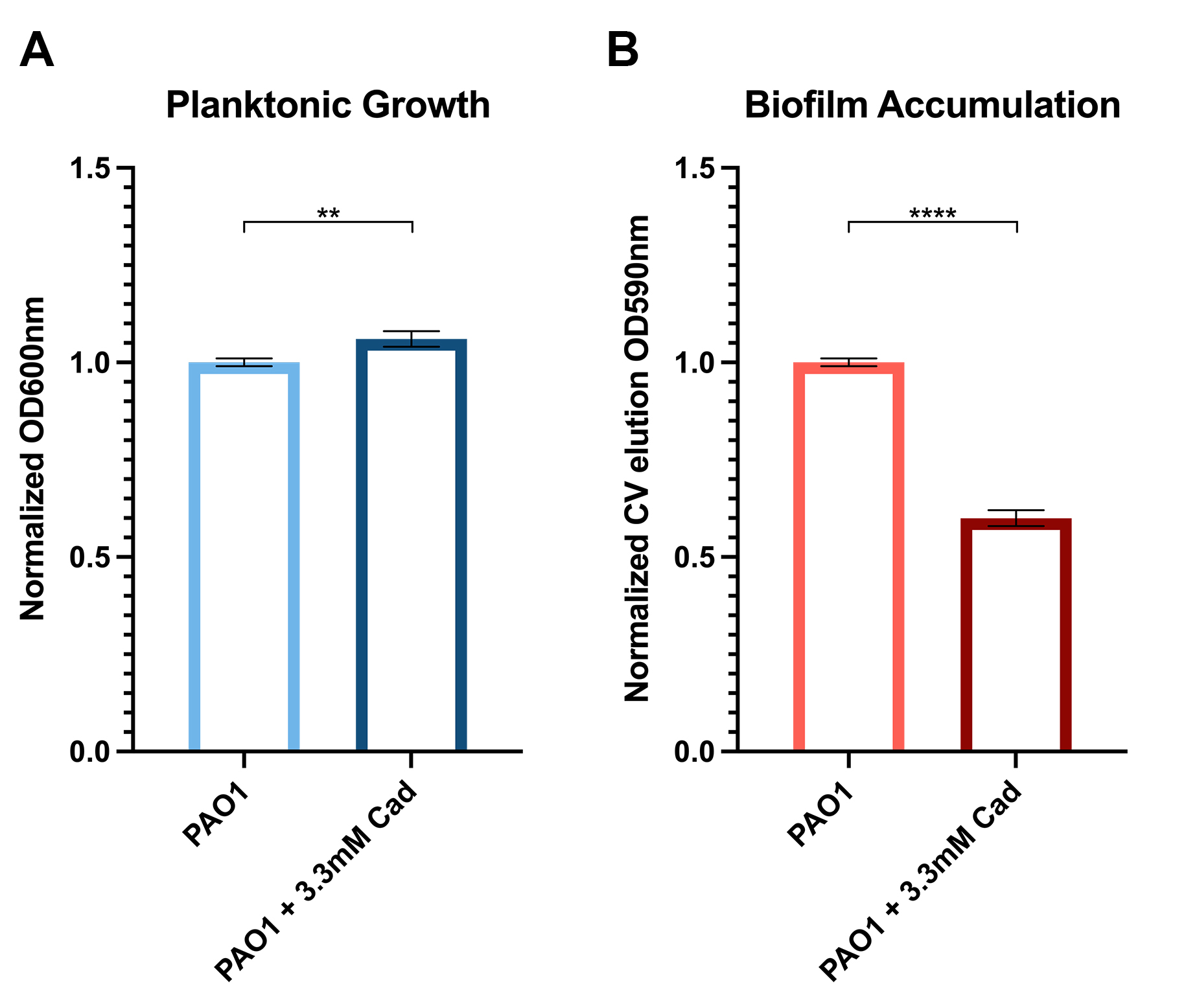

Supplement: Supplementary file 1 [file DataSheet_1.zip › Supplementary Material Presentation/SuppFig4.jpg]

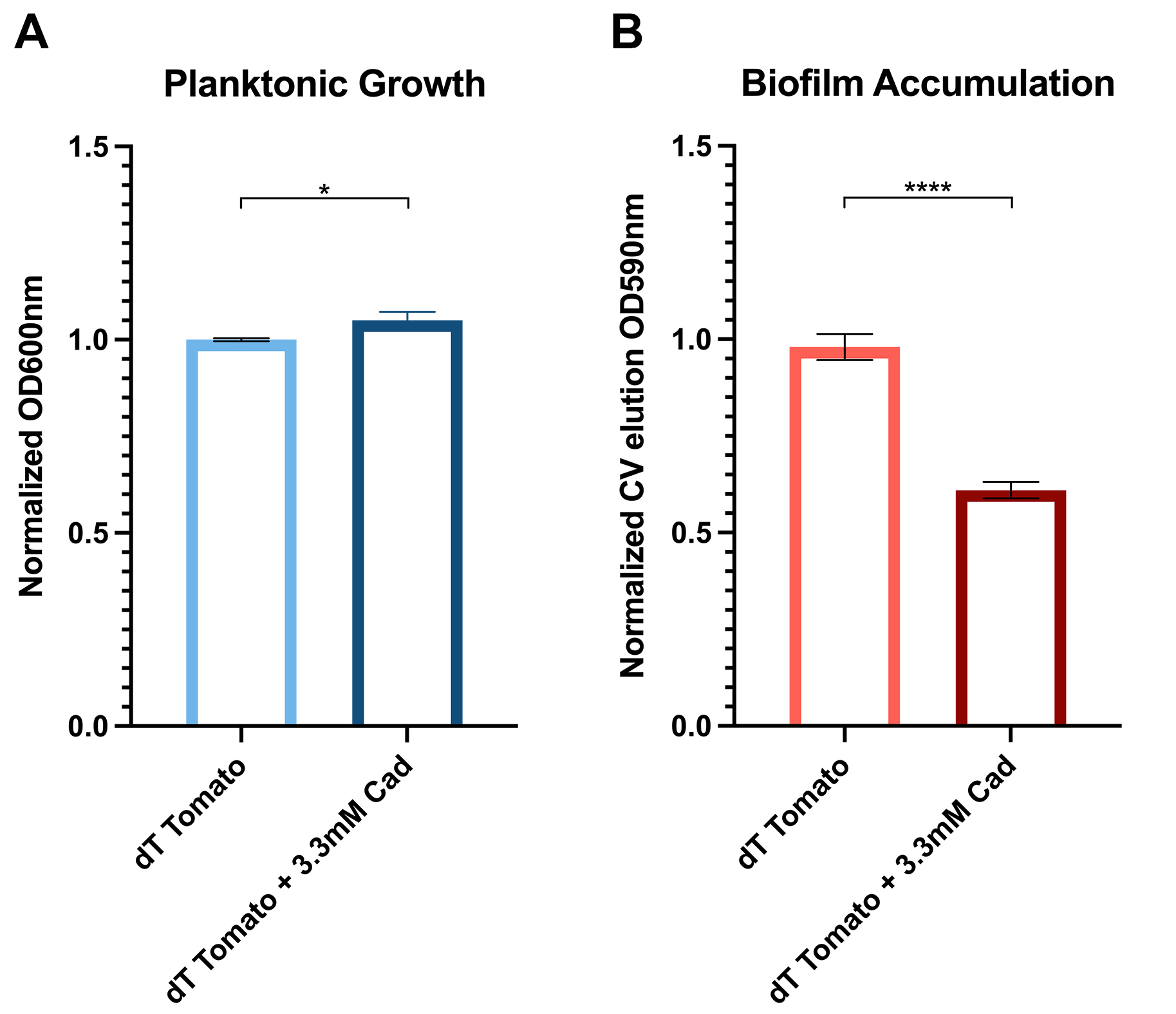

Supplement: Supplementary file 1 [file DataSheet_1.zip › Supplementary Material Presentation/SuppFig5.jpg]

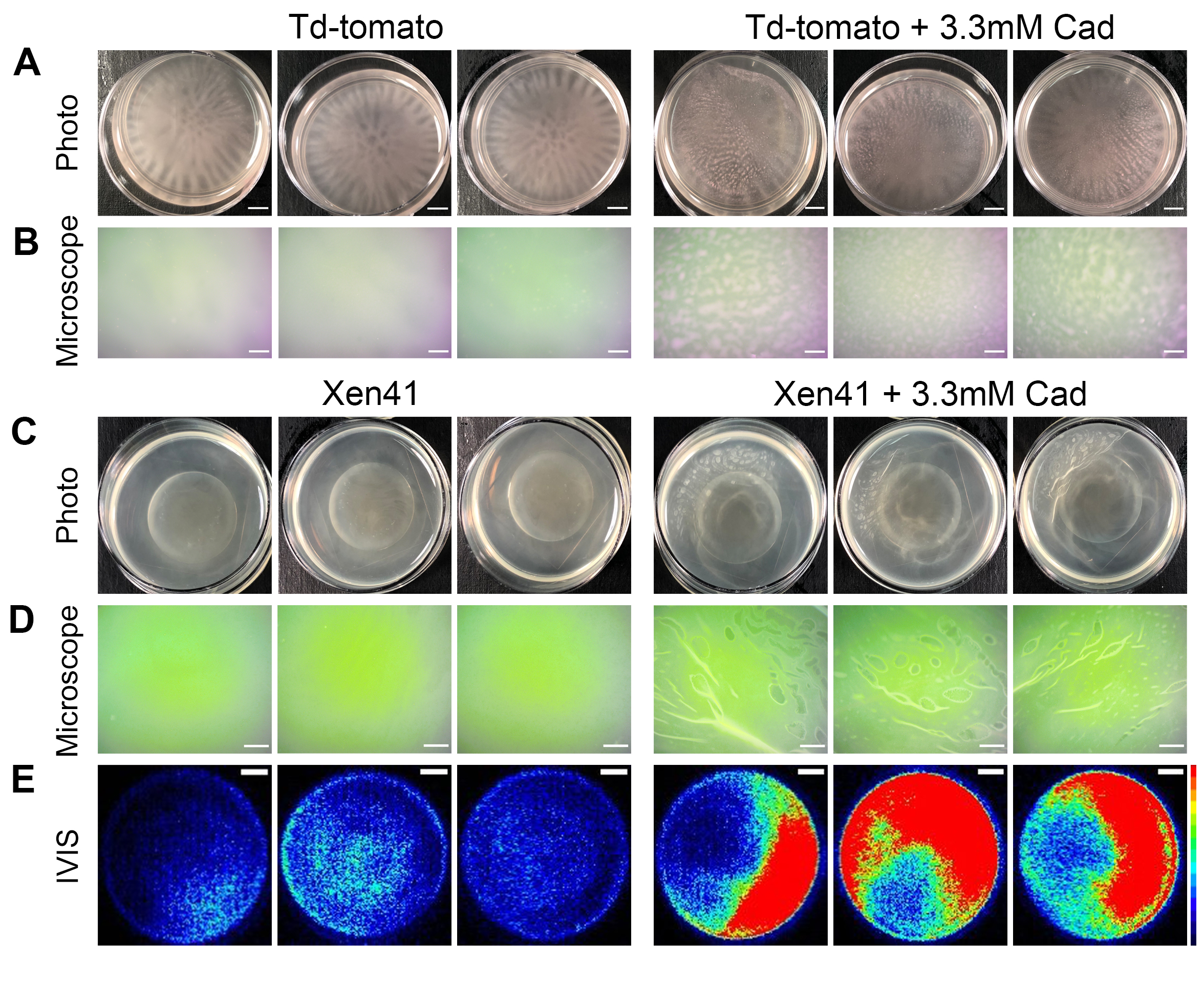

Supplement: Supplementary file 1 [file DataSheet_1.zip › Supplementary Material Presentation/SuppFig6.jpg]
