# Supplementary material for: Cadaverine Is a Switch in the Lysine Degradation Pathway in Pseudomonas aeruginosa Biofilm Identified by Untargeted Metabolomics
Source: Front Cell Infect Microbiol. 2022 Feb 14;12:833269. doi: 10.3389/fcimb.2022.833269 (PMC8884266; doi:10.3389/fcimb.2022.833269)
Supplement: Supplementary file 2 [file DataSheet_2.pdf]

Supplementary Material

Supplementary Figures

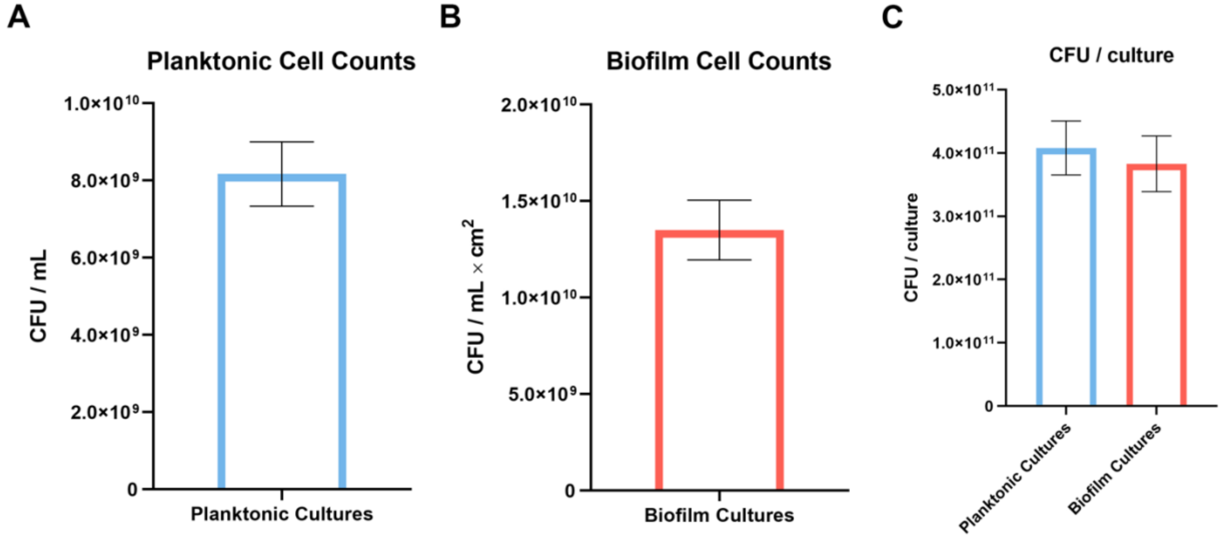

Supplementary Figure 1. Similar cell numbers as determined by CFUs were cultured for the biofilm and planktonic samples for the untargeted metabolomics analysis. Colony-forming units (CFUs)/mL for planktonic cultures (blue) (**A**) (n=6) and CFUs/mL × cm^2 for biofilm lawns (red) (**B**) (n=4) were measured by serial plate dilutions. When converted to CFU/culture (**C**) the cell count for planktonic and biofilm is 4.1×10^{11} and 3.8×10^{11}, respectively, which is not significantly different by unpaired, two-tailed *t*-test.

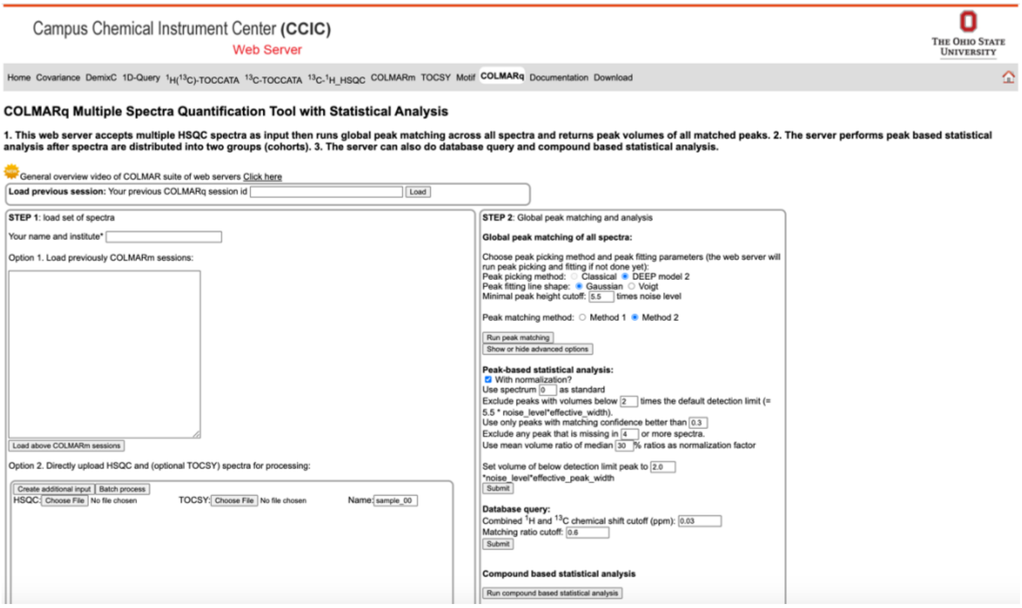

Supplementary Figure 2. Screenshot of the new public COLMARq web server user interface for metabolite identification and quantification that was utilized for the metabolomics NMR data analysis in this work. The ***co***mp***l***ex ***m***ixture ***a***nalysis by NM***R*** ***q***uantification web server allows accurate and efficient data analysis in a semi-automated manner. It allows the user to upload cohorts of 2D ^{13}C-^{1}H HSQC and 2D ^{1}H-^{1}H TOCSY spectra to perform the following tasks in a user-friendly manner: (i) semi-automated peak picking, (ii) peak fitting to obtain exact peak volumes for quantification, (iii) peak matching across multiple spectra, (iv) peak volume normalization via average median ratios, (v) querying against the COLMAR database containing over 750 reference spectra of hydrophilic metabolites for metabolite identification, (vi) performing uni- or multi-variate statistical analysis (PCA) in a cross-peak or metabolite-based manner, (vii) saving of existing jobs and loading of previous jobs to resume analysis, and (viii) downloading of COLMARq results for additional analysis using a user's preferred software and for publication. The COLMARq web server is publicly accessible at http://spin.ccic.osu.edu/index.php/quan/index/.

A

LB media pH with Cadaverine Supplementation	
pH	Cadaverine (μM)
6.89	0
7.09	300
7.50	900
7.71	3300

B

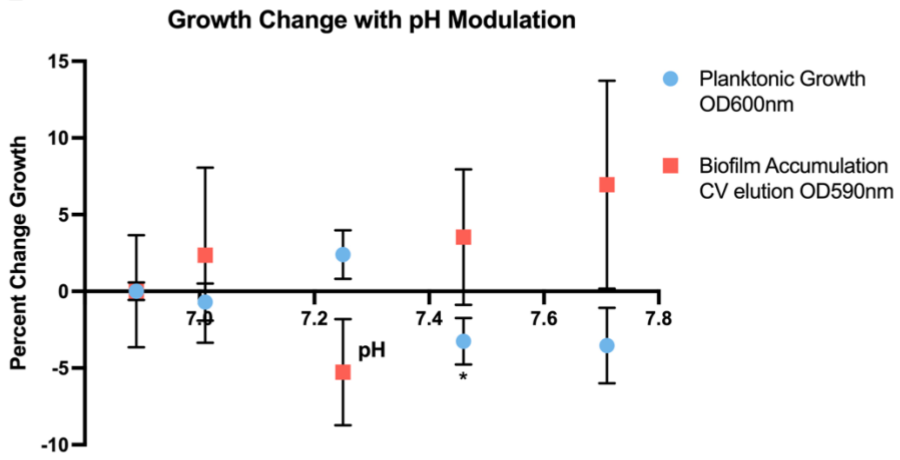

Supplementary Figure 3. Microtitre plate assays show cadaverine supplementation to the growth media slightly raises the pH, but this change does not cause a systematic significant change in planktonic growth or biofilm accumulation. pH measurement shows cadaverine supplementation raises the pH of the growth media up to 0.82 pH units with 3,300μM cadaverine (**A**). Planktonic growth (blue circle) and biofilm accumulation (red square) is measured by OD_{600} and crystal violet staining elution at OD_{590}, respectively, after raising the pH of the growth media from 6.9-7.7 with sodium hydroxide (NaOH) for 24 hrs (n=15). Values are normalized to the control wells (no NaOH) in each plate and reported as a percent change from control and asterisks denote significance (**B**) (* $p<0.05$ by unpaired, two-tailed *t*-test). Only at pH 7.5 planktonic growth shows a significant decrease ($p=0.048$) compared to the control. pH change causes no consistent significant change in planktonic growth and no significant change in biofilm accumulation.

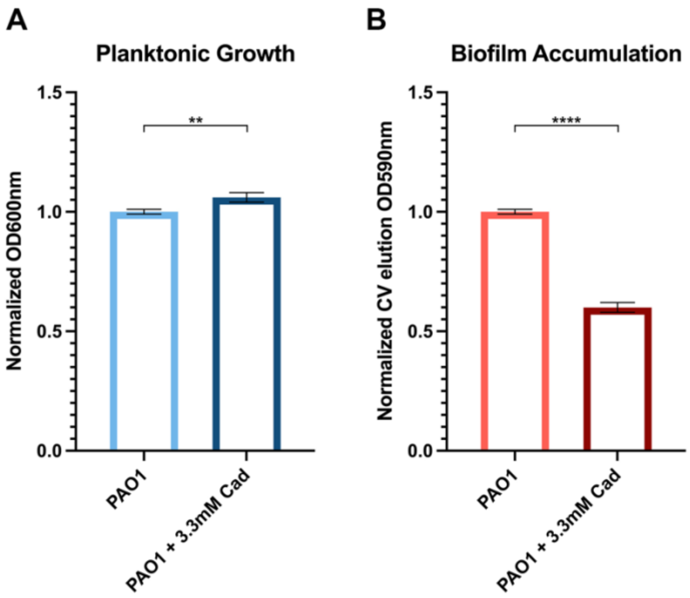

Supplementary Figure 4. Microtitre plate assays show cadaverine supplementation significantly increases planktonic growth and decreases biofilm accumulation in the presence of pre-formed biofilm. Planktonic growth (**A**) and biofilm accumulation (**B**) is measured by OD_{600} and crystal violet staining elution at OD_{590}, respectively, after growth for 24 hrs then supplementation of 0-3.30mM cadaverine (cad) to the growth media for an additional 24 hrs (n=100). Values are normalized to the control wells (no cadaverine) in each plate and reported as a percent change from control and asterisks denote significance (** p<0.01, **** p<0.0001 by unpaired, two-tailed t-test). (**A**) Planktonic growth significantly increases with cadaverine by 6%. (**B**) Biofilm accumulation significantly decreases with cadaverine by 40%.

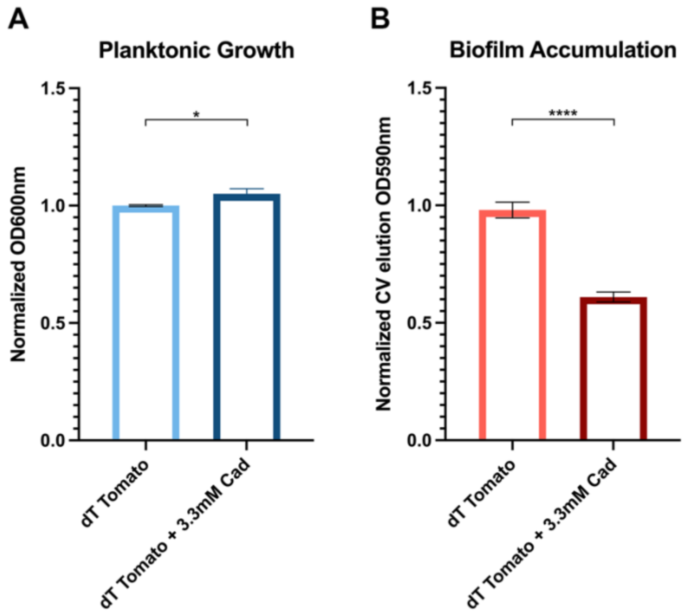

Supplementary Figure 5. Microtitre plate assays show cadaverine supplementation significantly increases planktonic growth and significantly decreases biofilm accumulation in PAO1 Td-tomato. Planktonic growth (**A**) and biofilm accumulation (**B**) is measured by OD_{600} and crystal violet staining elution at OD_{590}, respectively, after supplementation of 0-3.30mM cadaverine (cad) to the growth media for 24 hrs (n=75). Values are normalized to the control wells (no cadaverine) in each plate and reported as a percent change from control and asterisks denote significance (* p<0.05, **** p<0.0001 by unpaired, two-tailed t-test). (**A**) Planktonic growth significantly increases with cadaverine by 4%. (**B**) Biofilm accumulation significantly decreases with cadaverine by 37%. The PAO1 Td-tomato strain shows similar trends to the WT as seen in Figure 5.

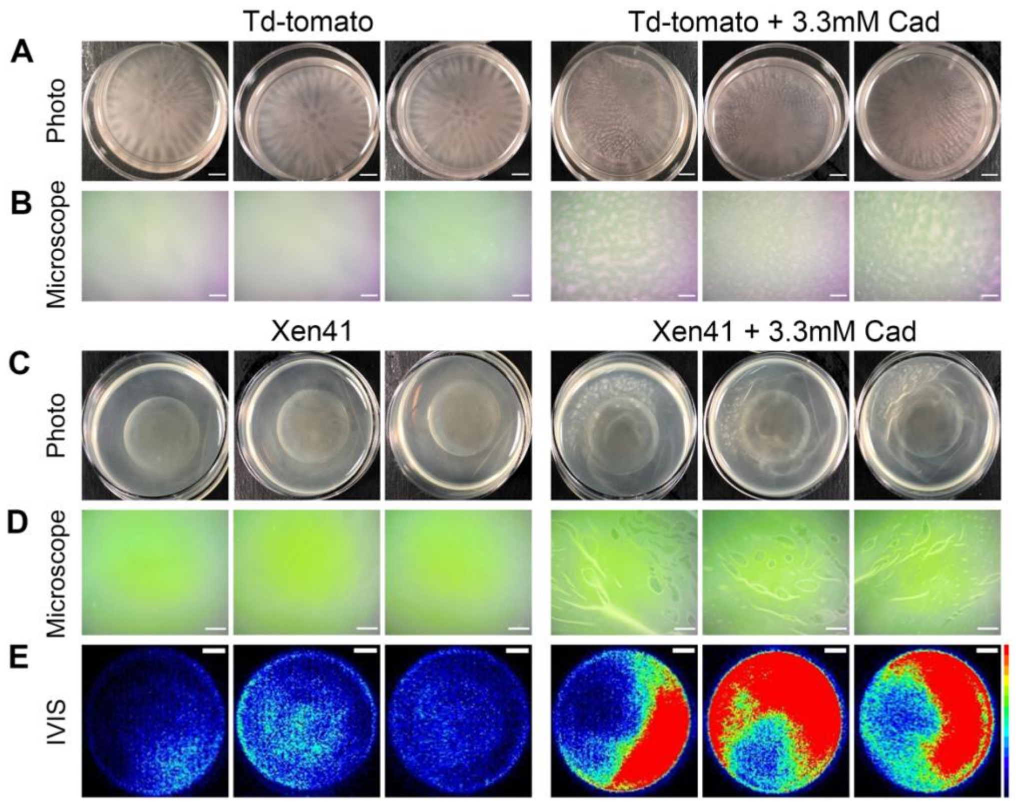

Supplementary Figure 6. Three replicates of images showing cadaverine supplementation causes altered biofilm morphology to pellicle biofilm at the air-liquid interface that is metabolically active. iPhone8 photos (scale bar = 2.9mm (**A**) and dissecting microscope images of culture air-liquid interfaces (scale bar = 0.14mm) (**B**) are taken of PAO1 Td-tomato supplemented with 3.30mM cadaverine (cad) showing pellicle biofilm. iPhone8 photos (scale bar = 3.6mm) (**C**), dissecting microscope images of culture air-liquid interfaces (scale bar = 0.18mm) (**D**), and IVIS images of air-liquid interfaces (scale bar = 3.8mm) with red being the most metabolically active (**E**) are shown of PAO1 Xen41 supplemented with 3.30mM cadaverine. Cadaverine supplementation leads to biofilm formation at the air-liquid interface compared to the control, where biofilm is localized at the bottom of the dish.

Supplementary Table 1. Peak list of unknown HSQC peaks rank ordered by *p*-value. Statistical significance determined by unpaired, two-tailed t-test. Average peaks volumes (n=9) are zero if the peaks are present in less than three samples within a cohort.

(see excel file: SuppTable1.xlsx)

Supplementary Table 2. Metabolite matching list and analysis of all identified metabolites rank ordered by *p*-value. Analysis includes the average metabolite peak volume per cohort (n=9), standard deviation, fold change (biofilm/planktonic), p-value determined by unpaired, two-tailed t-test, and false discovery rate test results. Metabolites that have a fold change greater than two and $p<0.05$ are shown in the top 25 metabolite changes.

(see excel file: SuppTable2.xlsx)

Supplementary Table 3. Mass spectrometry was used as a secondary method to confirm detection of lysine degradation pathway (LDP) intermediates. A representative planktonic culture was diluted and direct injected into a Q Exactive Plus Orbitrap mass spectrometer in positive ion mode. All intermediates of the cadaverine branch of the LDP were detected with at least one common adduct with a mass error of <0.77ppm except for glutaric acid. All adducts reported had mass error <1.25 ppm.

Metabolite	Formula	Monoisotopic mass (g/mol)	Adducts	Intensity	Mass error (ppm)
Lysine	$C_6H_{14}N_2O_2$	146.105528	+1 H'	1.46E+05	0.51
			+1 ACNH'	8.61E+04	1.04
			+2 H'	1.20E+07	0.61
Cadaverine	$C_5H_{14}N_2$	102.115698	+1 H'	1.09E+06	1.15
			+1 ACNH'	1.15E+06	0.76
5-Aminopentanal	$C_5H_{11}NO$	101.084064	+1 H'	1.82E+06	0.94
			+1 ACNH'	6.19E+05	0.61
			+1 NH4'	7.48E+04	0.79
5-Aminopentanoic acid	$C_5H_{11}NO_2$	117.0789786	+1 H'	5.61E+05	1.24
			+1 ACNH'	5.49E+05	0.42
Glutarate semialdehyde	$C_5H_8O_3$	116.0473441	+1 H'	1.48E+04	1.03
			+1 NH4'	4.46E+04	0.71
